# Nutritional Regulation of Cardiac Metabolism and Function: Molecular and Epigenetic Mechanisms and Their Role in Cardiovascular Disease Prevention

**DOI:** 10.3390/nu18010093

**Published:** 2025-12-27

**Authors:** Lucia Capasso, Donato Mele, Rosaria Casalino, Gregorio Favale, Giulia Rollo, Giulia Verrilli, Mariarosaria Conte, Paola Bontempo, Vincenzo Carafa, Lucia Altucci, Angela Nebbioso

**Affiliations:** 1 Department of Precision Medicine, University of Campania “Luigi Vanvitelli”, Vico L. De Crecchio 7, 80138 Naples, Italy; lucia.capasso2@unicampania.it (L.C.); donato.mele@unicampania.it (D.M.); rosaria.casalino@studenti.unicampania.it (R.C.); gregorio.favale@unicampania.it (G.F.); giulia.rollo1827@gmail.com (G.R.); mariarosaria.conte@unicampania.it (M.C.); paola.bontempo@unicampania.it (P.B.); vincenzo.carafa@unicampania.it (V.C.); lucia.altucci@unicampania.it (L.A.); 2 Biogem, Molecular Biology and Genetics Research Institute, 83031 Ariano Irpino, Italy; giulia.verrilli@unicampania.it; 3 Program of Medical Epigenetics, Vanvitelli Hospital, 80138 Naples, Italy

**Keywords:** diet, cardiac function, molecular pathways, epigenetics, cardiovascular disease, nutrient signaling

## Abstract

**Background**: Cardiovascular diseases (CVDs) remain the leading cause of mortality worldwide and are strongly influenced by dietary habits. Beyond caloric intake, nutrients act as molecular signals that regulate cardiac metabolism, mitochondrial function, inflammation, and epigenetic remodeling. **Objectives:** This review aims to synthesize current evidence on how dietary patterns and specific nutritional interventions regulate cardiac metabolism and function through interconnected molecular and epigenetic mechanisms, highlighting their relevance for cardiovascular disease prevention. **Methods:** A narrative review of the literature was conducted using PubMed, Scopus, and Web of Science, focusing on studies published between 2006 and 2025. Experimental, translational, and clinical studies addressing diet-induced modulation of cardiac metabolic pathways, oxidative and inflammatory signaling, epigenetic regulation, and gut microbiota-derived metabolites were included. **Results:** The analyzed literature consistently shows that unbalanced diets rich in saturated fats and refined carbohydrates impair cardiac metabolic flexibility by disrupting key nutrient-sensing pathways, including AMP-activated protein kinase (AMPK), proliferator-activated receptor alpha (PPARα), mammalian target of rapamycin (mTOR), and sirtuin 1/peroxisome proliferator-activated receptor gamma coactivator 1-alpha (SIRT1/PGC-1α), leading to mitochondrial dysfunction, oxidative stress, chronic inflammation, and maladaptive remodeling. In contrast, cardioprotective dietary patterns, such as caloric restriction (CR), intermittent fasting (IF), and Mediterranean and plant-based diets, enhance mitochondrial efficiency, redox balance, and metabolic adaptability. These effects are mediated by coordinated activation of AMPK-SIRT1 signaling, suppression of mTOR over-activation, modulation of nuclear factor kappa-light-chain-enhancer of activated B cells (NF-κB) and nuclear factor erythroid 2-related factor 2 (Nrf2) pathways, and favorable epigenetic remodeling involving DNA methylation, histone modifications, and non-coding RNAs. Emerging evidence also highlights the central role of gut microbiota-derived metabolites, particularly short-chain fatty acids, in linking diet to epigenetic and metabolic regulation of cardiac function. **Conclusions:** Diet quality emerges as a key determinant of cardiac metabolic health, acting through integrated molecular, epigenetic, and microbiota-mediated mechanisms. Targeted nutritional strategies can induce long-lasting cardioprotective metabolic and epigenetic adaptations, supporting the concept of diet as a modifiable molecular intervention. These findings provide a mechanistic rationale for integrating personalized nutrition into cardiovascular prevention and precision cardiology, complementing standard pharmacological therapies.

## 1. Introduction

CVD remains the leading cause of death worldwide [1]. The pathogenesis and development of CVD are strongly influenced by modifiable lifestyle factors, particularly dietary habits, which control lipid and glucose metabolism, as well as systemic inflammation [2]. In this context, growing evidence indicates that alterations in cardiac energy metabolism are not merely secondary consequences of disease but key drivers of myocardial dysfunction and cardiovascular risk. In addition to its traditional role as an energy provider, diet acts as a powerful regulator of the intracellular signaling networks that orchestrate cardiac energy homeostasis. Nutrient availability, quality, and timing dynamically shape cardiomyocyte metabolism by activating nutrient-sensing pathways that integrate energetic, redox, and inflammatory signals. These include PPARα, AMPK, mTOR, and SIRT1/PGC-1α axes, among others, which regulate substrate utilization, mitochondrial biogenesis, and anabolic/catabolic balance [3]. Other signaling pathways, such as NF-κB, Nrf2, PI3K/Akt, and MAPK, also play a role in regulating oxidative stress and inflammatory mechanisms, and thus the nutrient surplus associated with structural and functional changes in the heart [4]. While these pathways have been extensively studied individually, their coordinated regulation by dietary factors remains incompletely understood. New findings suggest that dietary and microbiota metabolites are also epigenetic modulators, controlling DNA methylation, histone acetylation, and non-coding RNAs, which have long-term effects on the regulation of cardiac genes and metabolism [5]. These epigenetic mechanisms provide a molecular memory of nutritional exposures, potentially linking early or chronic dietary patterns to long-lasting alterations in cardiovascular phenotype. Despite significant progress made to date, metabolic and epigenetic frameworks are not yet fully integrated into a single, unified field within nutritional cardiology. As a result, the current literature lacks an overarching synthesis that connects nutrient-driven metabolic signaling with epigenetic regulation of cardiac function. This review highlights nutritional approaches that improve metabolic flexibility and support cardioprotection, such as CR, IF, and the Mediterranean diet. The rationale for this review is to integrate molecular, metabolic, and epigenetic evidence into a cohesive framework that reflects the multidimensional impact of nutrition on the heart. It summarizes current research on how diet influences cardiac metabolism and function through molecular and epigenetic mechanisms, with a primary focus on mechanistic and translational studies, while also discussing potential clinical implications. Nutritional stimuli can impart long-lasting epigenetic modifications that maintain cardioprotective gene expression even after metabolic normalization [6]. By defining the mechanistic links between dietary patterns, intracellular signaling, and epigenetic remodeling, this review aims to provide added value for basic researchers, clinicians, and nutrition-focused healthcare professionals. Elucidating these nutritionally dependent pathways provides a conceptual framework for understanding how specific dietary patterns influence cardiac metabolism, oxidative homeostasis, and overall cardiovascular resilience.

## 2. Methods

### 2.1. Literature Search

To enhance transparency and reproducibility, this narrative review was informed by a structured literature search. PubMed, Scopus, and Web of Science were queried from 2006 until 2025, consistent with the earliest and latest items reported in the reference list. The literature search was designed to reflect the scope of the manuscript, focusing on the mechanistic links between diet and nutritional interventions, cardiac metabolism, and epigenetic regulation, while also considering the contribution of the gut–heart axis. Searches were performed around a core keyword block, alone and in combination, following precise criteria. In brief, the search strategy combined terms related to (i) the heart (e.g., heart/cardiac/myocardial), (ii) metabolic and mitochondrial processes (e.g., metabolism/mitochondria), (iii) dietary patterns or interventions (e.g., diet, caloric restriction, fasting/intermittent fasting, Mediterranean diet, plant-based or ketogenic approaches), and (iv) epigenetic regulation (e.g., epigenetics, DNA methylation, histone modifications/acetylation, HDACs, sirtuins, and microRNAs). When a more targeted screening was required, the same core query was refined by adding terms related to major metabolic, oxidative, or inflammatory pathways (e.g., AMPK, mTOR, NF-κB, Nrf2). Finally, to cover the gut–heart axis, additional searches combined microbiota-related terms (gut microbiota/microbiome), key microbial metabolites (SCFAs and TMAO), and cardiac/epigenetic terms.

### 2.2. Inclusion and Exclusion Criteria

Inclusion criteria were (i) peer-reviewed original studies (human, animal, or in vitro) investigating the impact of dietary patterns, CR/IF, ketogenic approaches, bioactive nutrients, or microbiota-derived metabolites on cardiac metabolism and function and/or related molecular pathways (e.g., AMPK, mTOR, SIRT1/PGC-1α, Nrf2, NF-kB), with specific attention to epigenetic mechanisms (DNA methylation, histone modifications, and non-coding RNAs); and (ii) systematic reviews/meta-analyses to contextualize evidence or summarize clinical outcomes. Exclusion criteria were studies addressing only pharmacological interventions without a nutritional rationale, consistent with the scope of the manuscript, and non-peer-reviewed publication types, e.g., editorials, commentaries, or conference abstracts.

## 3. Diet and Cardiac Metabolism: Molecular Regulation and Metabolic Adaptation

**Figure 1:**
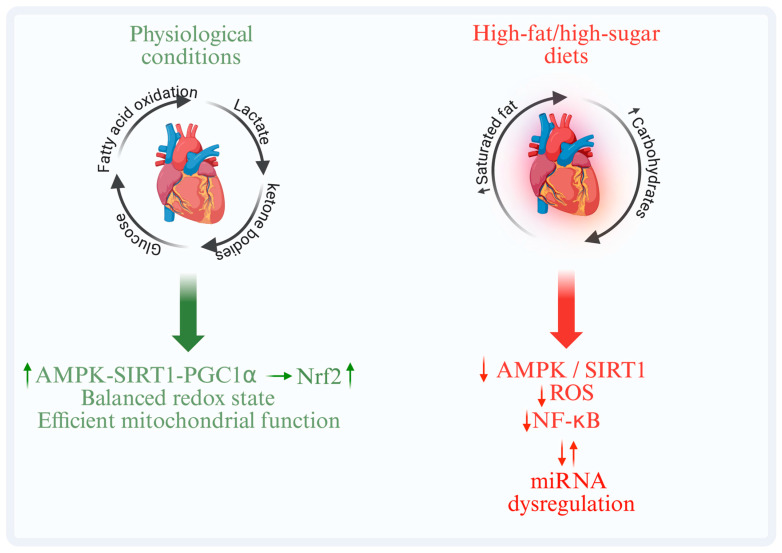
Cardiac metabolic flexibility under physiological and unbalanced dietary conditions. Balanced diets preserve cardiac metabolic flexibility through interconnected nutrient-sensing and redox pathways, including AMPK–SIRT1–PGC-1α and Nrf2. High-fat/high-sugar diets disrupt this network, promoting NF-κB-linked inflammatory and epigenetic signaling and maladaptive myocardial remodeling. ↑ indicates increase; ↓ indicates decrease. AMPK (AMP-activated protein kinase); SIRT1 (sirtuin1); PGC-1α (Peroxisome proliferator-activated receptor gamma coactivator 1-alpha); Nrf2 (nuclear factor erythroid 2-related factor 2); ROS (Reactive oxygen species); NF-κB (Nuclear factor kappa-light-chain-enhancer of activated B cells); miRNA (microRNA).

The heart’s highly adaptive metabolic network controls energy production in response to physiological and metabolic demands [7]. Fatty acid oxidation produces approximately 60–70% of myocardial adenosine triphosphate (ATP) under physiological conditions, while glucose, lactate, and ketone bodies maintain contractile efficiency [8]. Metabolic flexibility, the ability to dynamically switch between energy sources, is essential for maintaining bioenergetic homeostasis and ensuring optimal cardiac function under a wide range of physiological conditions [9]. Intracellular levels of acetyl-CoA and NAD^+^, cofactors required for chromatin-modifying enzymes and linking nutrient metabolism to cardiac gene regulation, are dynamically influenced by changes in substrate preference [10,11]. This flexibility is gradually compromised by nutritional overindulgence, particularly diets high in saturated fats or high-glycemic carbohydrates, which promote lipid overload, mitochondrial dysfunction, and excessive production of reactive oxygen species (ROS) [12]. Obesity, type 2 diabetes, and metabolic syndrome exacerbate these maladaptive processes, as dysregulation of hormonal and metabolic signaling accelerates structural remodeling and functional decline of the heart [13]. By stimulating nutrient-sensitive signaling pathways that improve oxidative capacity and redox balance, balanced dietary patterns such as the Mediterranean diet or CR, on the other hand, maintain metabolic flexibility and protect mitochondrial integrity [8,13]. Figure 1 illustrates this concept by comparing the negative consequences of unbalanced dietary patterns, high in fat and sugar, with physiological cardiac metabolic flexibility. Importantly, these processes do not occur in a linear sequence but are coordinated through an interconnected regulatory network. Nutrient-sensing pathways such as the AMPK–SIRT1–PGC-1α axis interact with redox regulators, including Nrf2, to sustain mitochondrial function and antioxidant defenses. In parallel, inflammatory signaling pathways, particularly NF-κB, are closely linked to epigenetic regulators such as miRNA networks, contributing to the amplification or resolution of diet-induced cardiometabolic stress.

### 3.1. Signaling Pathways and Molecular Regulators of Cardiac Metabolism

An integrated network of nutrient sensors and intracellular signaling pathways that synchronize substrate availability with myocardial metabolic needs maintains cardiac energy homeostasis. Among these regulatory systems, PPARα plays a pivotal role by controlling the transcription of genes involved in fatty acid β-oxidation. Activation of this pathway by unsaturated fatty acids or ketone bodies enhances lipid utilization and prevents the accumulation of lipotoxic intermediates, whereas chronic exposure to saturated fats leads to loss of metabolic flexibility and cardiac dysfunction [13]. AMPK acts as the main cellular energy sensor in conjunction with PPARα: it promotes substrate oxidation, mitochondrial biogenesis, and antioxidant defenses in response to an increased AMP/ATP ratio, ensuring an effective adaptive response to metabolic stress. In addition to its energy-sensing function, AMPK activation links nutrient sensing with mitochondrial and transcriptional regulation by promoting SIRT1-PGC-1α-dependent chromatin remodeling [14,15]. However, prolonged caloric overload reduces its activity, compromising energy stability and cardiac metabolic efficiency [16]. mTOR functions as a complementary nutrient-sensing anabolic regulator: when this pathway is continuously activated during nutrient overload, it promotes cell growth and protein synthesis but gradually causes maladaptive cardiac hypertrophy. On the other hand, CR rebalances anabolic and catabolic processes [17]. The SIRT1/PGC-1α axis completes this regulatory circuit by integrating energy and redox pathways to improve oxidative efficiency and modulate mitochondrial gene expression. Polyphenols and omega-3 fatty acids are examples of bioactive nutrients that can positively impact the AMPK-SIRT1-PGC1α axis through complementary mechanisms. Polyphenols, such as resveratrol and flavonoids, activate AMPK and SIRT1 either directly or indirectly by altering cellular redox and NAD^+^ availability, thereby enhancing mitochondrial biogenesis, oxidative metabolism, and antioxidant defenses. Omega-3 fatty acids (e.g., EPA and DHA) improve mitochondrial function by reshaping membrane lipid composition, regulating lipid-derived signaling mediators, and attenuating inflammatory and oxidative stress pathways. Together, these effects contribute to improved metabolic homeostasis, preservation of cardiac energetic efficiency, and protection against maladaptive cardiac remodeling [18,19]. An overview of the major signaling pathways and molecular regulators governing cardiac metabolic control is provided in Table 1.

### 3.2. Interplay Between Metabolism, Inflammation, and Oxidative Stress

Inflammatory and oxidative pathways are triggered by metabolic imbalance caused by nutrient overload and reduced metabolic flexibility, which progressively compromises cardiac function. Excessive consumption of simple sugars and saturated fats activates the NF-κB and MAPK signaling pathways, which in turn stimulate cytokine production, immune cell recruitment, and fibrotic remodeling of the heart [20]. Nutritional excess itself weakens antioxidant defenses and causes the accumulation of ROS and mitochondrial damage by suppressing AMPK and SIRT1, important regulators of cellular energy homeostasis and redox balance [21]. Conversely, metabolites and bioactive substances such as polyphenols and short-chain fatty acids (SCFAs) from the gut microbiota and diet have complementary protective effects. These substances improve redox homeostasis and reduce inflammation-induced metabolic damage by activating Nrf2 and other cytoprotective pathways [22]. In this context, AMPK-SIRT1-PGC-1α signaling and Nrf2-mediated antioxidant defenses do not act in isolation but form an integrated cytoprotective module, whereby activation of nutrient-sensing pathways supports Nrf2-driven detoxification and redox balance. This reciprocal regulation further supports the concept of cardiometabolic control as a dynamic and interconnected network rather than a unidirectional cascade. Therefore, healthy dietary habits, particularly the Mediterranean diet and CR, reduce inflammation and oxidative stress, maintain contractile function, and halt maladaptive cardiac remodeling [23].

## 4. Diet, Inflammation and Oxidative Stress

The redox and inflammatory pathways that regulate cardiac function are directly influenced by changes in diet composition and quality. A diet high in saturated fats and simple sugars and low in protective nutrients promotes fibrosis, structural remodeling, and decreased reserve (pumping ability) of heart [24,25,26]. This condition maintains chronic low-grade inflammation, which increases cardiovascular susceptibility and promotes the development of cardiometabolic disorders [27,28,29].

### 4.1. Activation of Inflammatory and Redox Pathways

**Figure 2:**
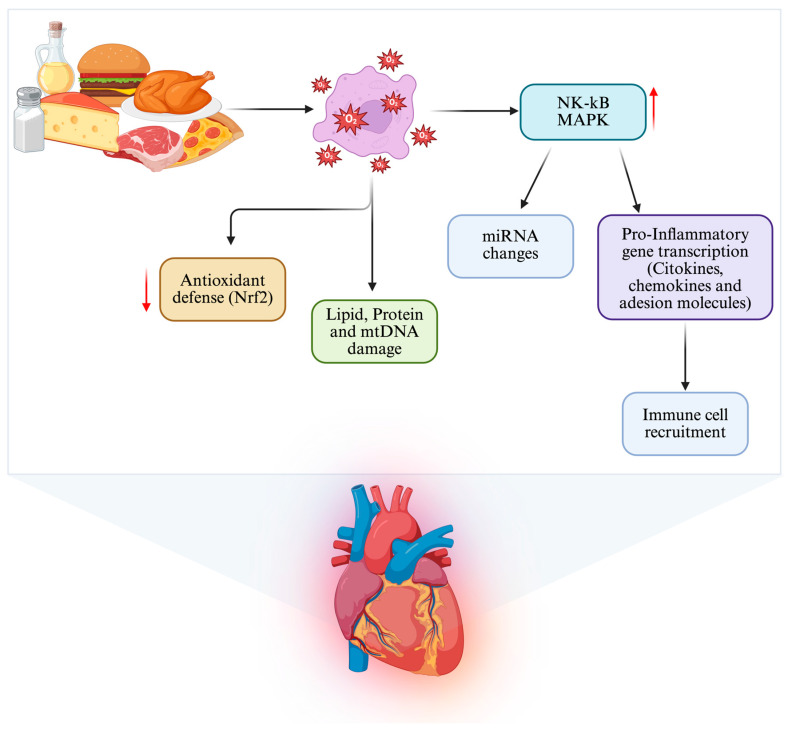
Diet-induced oxidative, inflammatory, and epigenetic stress in the heart. Unhealthy dietary patterns activate interconnected metabolic, inflammatory, oxidative, and epigenetic pathways that converge to promote cardiometabolic stress and cardiac remodeling. NF-κB (Nuclear factor kappa-light-chain-enhancer of activated B cells); MAPK (Mitogen-activated protein kinase); Nrf2 (nuclear factor erythroid 2-related factor 2); miRNA (microRNA).

Significant consumption of saturated fats and simple sugars is a potent pro-inflammatory factor in the cardiovascular system, triggering complex intracellular signaling pathways in endothelial cells, cardiomyocytes, and resident leukocytes [30,31]. Overnutrition increases the production of ROS at both the mitochondrial and cytosolic levels, resulting in altered redox balance and activation of oxidative stress-sensitive transcription factors, including NF-κB and MAPK. These pathways enhance the transcription of pro-inflammatory genes encoding cytokines, chemokines, and adhesion molecules, thus contributing to the recruitment of immune cells and the progression of fibrotic processes [32,33]. At the same time, impaired activation of adaptive antioxidant defenses, particularly those regulated by Nrf2, reduces cellular resilience to oxidative stress, resulting in the accumulation of lipid peroxidation products, protein carbonyls, and mitochondrial DNA damage [34,35]. Excessive caloric intake alters signaling through pathways including PI3K/Akt and MAPK, exacerbating inflammation, endothelial dysfunction, and the gradual deterioration of coronary reserve and contractile function [36,37]. These processes collectively outline an integrated framework in which diet and oxidative stress function as interdependent elements of a metabolic–inflammatory dysregulation circuit that contributes to cardiovascular susceptibility (Figure 2).

### 4.2. Interaction Between Chronic Inflammation and Cardiac Remodeling

Chronic low-grade inflammation is an important pathophysiological mechanism linking diet to cardiac problems. Chronic inflammation differs from the acute, temporary, and functional inflammatory response required to restore the body to normal. In chronic inflammation, pro-inflammatory mediators are constantly activated, which alters intercellular signaling between endothelial cells, cardiomyocytes, and resident immune cells. This inflammatory microenvironment maintains oxidative stress and metabolic dysfunction, thus exacerbating cardiac susceptibility and accelerating tissue damage [38,39]. Prolonged activation of NF-κB and MAPK signaling causes maladaptive remodeling, resulting in cardiomyocyte hypertrophy, extracellular matrix accumulation, and interstitial fibrosis, which increase myocardial stiffness and impair diastolic function [40,41]. Furthermore, endothelial dysfunction is intensified by reduced nitric oxide (NO) bioavailability and the overexpression of adhesion molecules, which highlight the mismatch between myocardial oxygen supply and demand. The result, especially in patients with obesity, type 2 diabetes, or metabolic syndrome, is a vicious cycle of oxidative stress, structural remodeling, and energy dysfunction that promotes progression from subclinical impairment to full-blown heart failure [42,43,44].

### 4.3. Modulatory Role of Bioactive Nutrients

Cellular pathways integrating metabolism, inflammation, and oxidative stress are actively modulated by a variety of nutrients and dietary patterns, which directly impact cardiac function and functional integrity. By inhibiting Nrf2 activity and persistently activating NF-κB, high-fat or high-carbohydrate diets impair these regulatory networks and prolong inflammation and intracellular oxidation [45,46]. This imbalance directly impacts myocardial contractility and energy efficiency, promoting lipotoxicity, mitochondrial dysfunction, and dysregulated gene transcription [47]. On the other hand, specific nutritional interventions that reduce oxidative stress and promote more effective metabolic remodeling include a low-carbohydrate diet, the Mediterranean diet, and dietary patterns rich in antioxidants, polyphenols, and unsaturated fatty acids. These interventions activate important signaling pathways such as AMPK, Nrf2, and SIRT1 [48,49,50]. Regular consumption of fiber and unsaturated fats also contributes to improved insulin sensitivity, endothelial function, and systemic inflammatory balance. Together, these findings highlight that diet should be regarded not merely as an energy source but as a true epigenetic and metabolic modulator, capable of directing key signaling pathways involved in cardiovascular health and guiding personalized preventive and therapeutic strategies [51]. The modulatory effects of bioactive nutrients on oxidative and inflammatory pathways are summarized in Table 2.

## 5. Diet and Epigenetics in the Regulation of Cardiac Function

Cardiac metabolism and functional integrity are modulated by epigenetic mechanisms, which form a dynamic interface between diet, environmental factors, and gene expression. Unlike genetic mutations, epigenetic modifications control transcriptional activity in a reversible and context-dependent manner, converting metabolic and nutritional signals into long-term cellular adaptations. The heart is particularly vulnerable to these mechanisms due to its high energy expenditure and constant exposure to systemic fluctuations. Nutrient availability and chromatin dynamics are mechanistically linked by metabolic intermediates such as acetyl-CoA, S-adenosylmethionine, and α-ketoglutarate, which directly fuel epigenetic enzymes [52]. The maintenance or loss of cardiac homeostasis is influenced by epigenetic remodeling, which affects mitochondrial efficiency, substrate utilization, and stress response [53,54].

### 5.1. DNA Methylation and Cardiac Gene Regulation

**Figure 3:**
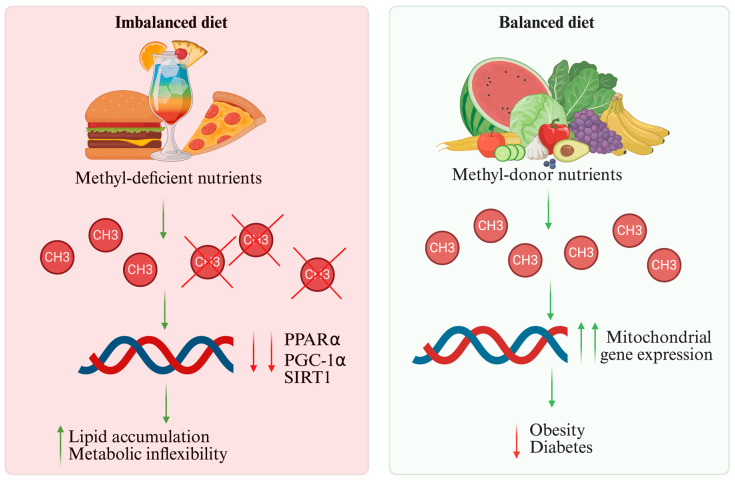
Epigenetic effects of dietary methyl donors on cardiac metabolism. Methyl-deficient diets reduce DNA methylation of key mitochondrial genes, impairing oxidative metabolism and metabolic flexibility. In contrast, diets rich in methyl donors restore proper methylation, enhance mitochondrial gene expression, and reduce cardiometabolic risk. ↑ indicates increase; ↓ indicates decrease.PPARα (Peroxisome proliferator-activated receptor alpha); PGC-1α (Peroxisome proliferator-activated receptor gamma coactivator 1-alpha); SIRT1 (Sirtuin1).

One of the main ways diet influences gene expression in the heart is through DNA methylation. DNA methyltransferases (DNMTs) add methyl groups to DNA cytosines in this process, resulting in transcriptional modulation or gene silencing. The methylation profile of important mitochondrial metabolism genes, such as PPARα, PGC-1α, and SIRT1, can be altered by unbalanced diets, particularly those high in saturated fat and low in methyl-donor nutrients such as folate and betaine. This can promote lipid accumulation and reduce metabolic flexibility [55,56]. On the other hand, healthy dietary habits rich in methyl donors such as choline, methionine, and folate improve mitochondrial gene expression, restore proper methylation, and reduce the risk of cardiometabolic dysfunction related to obesity and diabetes [57,58] (Figure 3). SCFAs and other gut microbiota-derived metabolites, including tryptophan-derived AhR ligands, B-group vitamins, and polyamines, act as cofactors for epigenetic enzymes and therefore exert a significant regulatory influence. They directly influence DNMT activity and modulate the availability of methyl substrates, creating a functional gut–heart axis that links nutrition to epigenetic regulation [58]. One-carbon metabolism coordinates the balance of methylation with mitochondrial oxidative capacity by integrating DNMT activity with dietary methyl donors such as folate and choline [59]. Figure 3 summarizes these connections by showing how methyl-deficient nutrients and dietary methyl donors influence mitochondrial gene expression and cardiac DNA methylation.

### 5.2. Histone Modifications and Control of Transcriptional Expression

**Figure 4:**
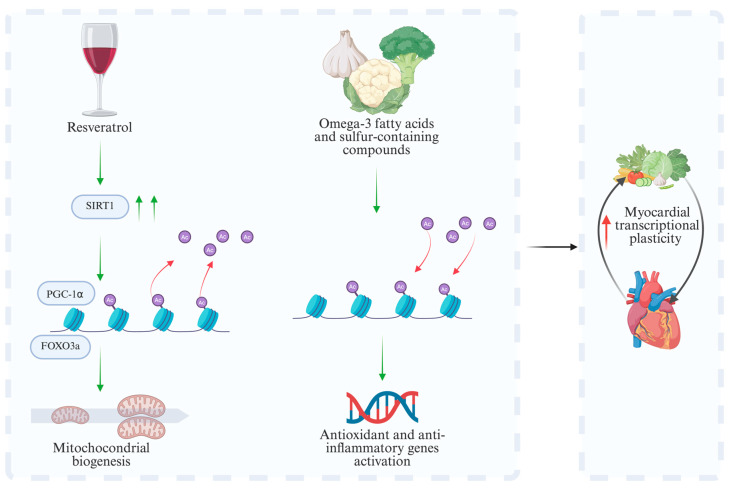
Epigenetic effects of nutrients on myocardial transcriptional plasticity. Nutrients such as resveratrol, omega-3 fatty acids, and sulfur-containing compounds modulate myocardial epigenetic pathways, enhancing mitochondrial biogenesis, antioxidant gene expression, and cardiac adaptive capacity under metabolic and oxidative stress. Green and red arrows indicate modulation or changes in biological processes; black arrows indicate downstream functional outcomes. SIRT1 (Sirtuin1); PGC-1α (Peroxisome proliferator-activated receptor gamma coactivator 1-alpha); FOXO3a (Forkhead box O3a).

Histone acetylation and deacetylation constitute a key epigenetic mechanism through which diet modulates chromatin structure and transcriptional activity. Histone acetyltransferases (HATs) promote chromatin relaxation and transcriptional activation, whereas histone deacetylases (HDACs) and sirtuins, particularly SIRT1, function as metabolic sensors that coordinate nutritional and redox responses [60]. Resveratrol, a dietary polyphenol found in red wine, activates SIRT1, leading to the deacetylation of PGC-1α and Forkhead box O3a (FOXO3a), and promotes mitochondrial biogenesis and resistance to oxidative stress [61,62]. Likewise, omega-3 fatty acids and sulfur-containing compounds from garlic and cruciferous vegetables modulate HDAC activity, enhancing chromatin accessibility and promoting the expression of antioxidant and anti-inflammatory genes [63,64]. Together, these epigenetic adaptations sustain redox balance and mitochondrial integrity, reinforcing the connection between diet and myocardial transcriptional plasticity. These mechanisms are illustrated in Figure 4, which summarizes how specific nutrients modulate histone acetylation and deacetylation to regulate mitochondrial biogenesis, antioxidant defenses, and myocardial transcriptional flexibility.

### 5.3. Regulation of Non-Coding RNAs and Epigenetic Remodeling

Diet influences gene expression through non-coding RNAs (ncRNAs), which control transcriptional and post-transcriptional processes, as well as DNA and histone modifications. Among these, long non-coding RNAs (lncRNAs) and miRNAs are essential for coordinating oxidative stress and inflammatory responses. The cardiac miRNA profile is significantly altered by dietary patterns high in saturated fats and simple sugars, which increase pro-inflammatory and pro-fibrotic signaling. Apoptotic and fibrotic pathways have been linked to increased expression of miR-21 and miR-34a, resulting in myocardial remodeling and reduced contractile function [65,66]. NF-κB functions as a key upstream regulator of several of these miRNAs and, in turn, is itself modulated by miRNAs that buffer or amplify inflammatory signaling, underscoring the bidirectional cross-talk between NF-κB pathways and ncRNA networks in diet-induced cardiac remodeling [67]. Notably, this relationship is organized as a feedback circuit: NF-κB can promote the transcription of inflammation- and fibrosis-associated miRNAs, while specific miRNAs can restrain or potentiate NF-κB activity, thereby influencing the magnitude and persistence of inflammatory and remodeling responses to dietary stress. Conversely, following a Mediterranean diet, rich in polyphenols, fiber, and unsaturated fatty acids, increases the expression of miR-133a and miR-499, both involved in maintaining contractility and protecting cardiomyocytes [68,69,70]. Some nutrients, particularly methyl-donor substrates such as folate, choline, methionine, and betaine, as well as polyphenols and long-chain omega-3 fatty acids, act as natural epigenetic modulators along these pathways, helping to counteract the detrimental effects of high-calorie diets and to restore cardiac transcriptional balance (mainly summarized in Table 2 and, at the level of dietary patterns, in Table 3). Overall, DNA methylation, histone remodeling, and ncRNA regulation form an integrated epigenetic network through which diet orchestrates myocardial gene expression and long-term adaptive responses. Thus, the cardiac epigenome appears as a precisely tuned interface between genomic function and the nutritional environment. Diet has long-term effects on mitochondrial metabolism, redox balance, and cellular integrity by altering methylation patterns, chromatin architecture, and RNA-mediated signaling. These mechanisms demonstrate the potential of nutritional strategies that target epigenetics to prevent or mitigate cardiometabolic diseases. Precision nutritional approaches, linking molecular prevention and personalized cardiology by tailoring dietary interventions to individual epigenetic profiles, could become possible if this knowledge is integrated into clinical practice. An overview of the key dietary factors that alter epigenetic pathways to promote cardiac protection is provided in Table 3.

### 5.4. Nutrient-Dependent Epigenetic Modulation of Cardiac Gene Programs

Diet quality and micronutrient sufficiency are key determinants of the cardiac epigenome, as they provide both substrates and cofactors required by enzymes controlling DNA and histone methylation/demethylation. Adequate intake of methyl donors (folate, choline, betaine, and methionine) together with one-carbon pathway cofactors (B vitamins, particularly B2, B6, and B12) supports S-adenosylmethionine availability and DNMT activity, helping maintain appropriate methylation patterns in genes governing mitochondrial biogenesis and oxidative defense (e.g., PGC-1α and SIRT1). In parallel, TET-dependent demethylation relies on additional cofactors such as iron (Fe^2+^), α-ketoglutarate, and vitamin C, further strengthening the link between micronutrient adequacy and epigenetic plasticity [56,57,58,59,60]. Moreover, bioactive nutrients and microbiota-derived metabolites that influence histone acetylation (polyphenols, omega-3 fatty acids, and SCFAs) promote chromatin states associated with enhanced antioxidant capacity, improved mitochondrial efficiency, and attenuation of inflammatory signaling [60,75,76,77]. Through these nutritionally regulated epigenetic mechanisms, epigenetic regulation is translated into functional cardiac gene programs, oxidative defense and mitochondrial biogenesis, supporting metabolic flexibility, myocardial resilience, and long-term cardioprotection.

## 6. Cardioprotective Nutritional Strategies

Certain dietary approaches can preserve myocardial health through metabolic reprogramming, based on growing knowledge of the epigenetic mechanisms linking nutrition and cardiac metabolism. By restoring the molecular coordination between energy production, oxidative balance, and cellular resilience, cardioprotective nutrition goes beyond reducing traditional risk factors. These interventions improve metabolic flexibility and promote long-term cardiac fitness by precisely adjusting caloric intake, nutrient composition, and meal timing [78,79].

### 6.1. CR and IF

**Figure 5:**
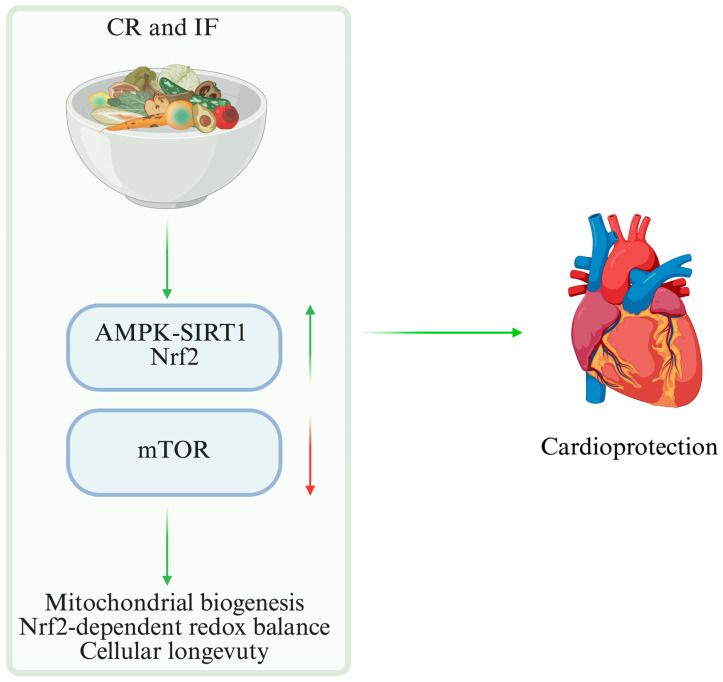
Effects of CR and IF on cardiac metabolism. CR and IF transiently activate AMPK-SIRT1 and inhibit mTOR, promoting mitochondrial biogenesis, redox balance, and enhanced cellular stress resistance. These coordinated metabolic and epigenetic adaptations contribute to long-term cardioprotection. Green arrows indicate activation or upregulation of pathways, whereas red arrows indicate downregulation of signaling. CR (Calorie Restriction); IF (Intermittent Fasting); AMPK (AMP-activated protein kinase); SIRT1 (Sirtuin1); Nrf2 (nuclear factor erythroid 2-related factor 2); mTOR (Mammalian target of rapamycin).

Nutritional strategies like CR and IF modify cardiac metabolism by coordinating metabolic and epigenetic processes. These interventions function as physiological modulators in addition to lowering calorie intake; they temporarily activate AMPK-SIRT1 signaling and inhibit mTOR, which promotes mitochondrial biogenesis, redox balance, and cellular longevity [80,81]. This adaptive response is further supported by cross-talk with Nrf2-dependent antioxidant pathways, reinforcing redox homeostasis and cardioprotection. Both CR and IF reduce systemic inflammation, improve substrate utilization, and improve myocardial performance and adaptive capacity under stress, according to experimental and clinical data [82,83]. The maintenance of cardiac homeostasis and long-term myocardial protection is ultimately facilitated by the integration of metabolic and nutritional signals, which offer promising models for nutritional therapy. These mechanisms are summarized in Figure 5, which illustrates how caloric restriction and IF regulate metabolic and epigenetic pathways to promote cardioprotection.

### 6.2. Mediterranean and Plant-Based Diets

Among the various nutritional models, the Mediterranean diet remains the most extensively validated approach for cardiovascular prevention. Its cardioprotective effect derives from a synergistic combination of monounsaturated fats, polyphenols, and fiber, which coordinate antioxidant, anti-inflammatory, and metabolic signaling through the activation of Nrf2, SIRT1, and PGC-1α [84,85]. By improving endothelial function, preserving lipid homeostasis, and reducing systemic inflammation, this diet sustains vascular integrity and myocardial efficiency [86]. In addition to these functional effects, recent studies indicate that the Mediterranean diet also promotes epigenetic stability, enhancing DNA methylation of cardioprotective genes and downregulating pro-inflammatory miRNA [74]. This coordinated molecular response contributes to long-term metabolic flexibility and protection against cardiac remodeling. Plant-based diets, characterized by a high intake of vegetables, whole grains, legumes, and nuts, share many of these beneficial signatures. Their efficacy relies on a dense supply of antioxidant phytocompounds, lower saturated fat intake, and modulation of the gut microbiota, leading to increased SCFAs and improved epigenetic–metabolic balance within the myocardium [75]. Together, these dietary patterns represent complementary paradigms of nutritional cardioprotection, integrating metabolic, vascular, and epigenetic mechanisms to maintain cardiac health. The comparative molecular effects of the Mediterranean and plant-based diets on cardiac metabolism (Table 4).

### 6.3. Bioactive Nutrients and Nutraceutical Supplementation

By regulating metabolic and epigenetic networks, numerous bioactive nutrients and nutraceuticals support cardioprotection, in addition to a well-rounded diet. In this context, the use of bioactive nutrients and nutraceutical supplementation denotes the targeted intake of specific compounds, such as polyphenols (e.g., resveratrol, quercetin, curcumin), long-chain omega-3 fatty acids (EPA and DHA), methyl-donor substrates (folate, betaine, choline), and mitochondrial- or autophagy-supporting agents (e.g., coenzyme Q10, spermidine), usually provided as standardized supplements alongside cardioprotective dietary patterns. These molecules can act synergistically with dietary interventions to enhance mitochondrial biogenesis, reduce inflammation, and maintain redox balance [73,87,88,89]. Consequently, they influence DNA methylation and miRNA profiles related to oxidative stress and myocardial fibrosis, influencing the activity of SIRT1, Nrf2, and other transcriptional regulators. Although available clinical studies differ in terms of doses, formulations, and duration, most reported benefits occur at intake levels compatible with high adherence to Mediterranean-like diets or with moderate supplemental doses, while the optimal regimen still needs to be defined in larger, well-controlled trials [90]. When integrated into an overall healthy nutritional framework and used with appropriate clinical judgment, these nutraceutical strategies may help improve cardiac function and enhance resistance to oxidative stress [86,91].

### 6.4. Vegetarian and Vegan Diets: Clinical Evidence and Mechanisms

Vegetarian and vegan dietary patterns, which exclude meat and, in the case of vegan diets, all animal-derived products, can be regarded as a more intensive form of plant-based eating compared with the traditional Mediterranean diet. Evidence from randomized trials and meta-analyses indicates that these diets are associated with reductions in total cholesterol, LDL cholesterol, and apolipoprotein B, as well as improvements in HbA1c and body weight, particularly among individuals with overweight/obesity, type 2 diabetes, or established cardiovascular risk [92,93,94,95,96]. From a mechanistic perspective, vegetarian and vegan diets combine low intakes of saturated fat and dietary cholesterol with high intakes of fiber, complex carbohydrates, unsaturated fatty acids, and polyphenol-rich plant foods (Table 5). This nutritional profile supports improved insulin sensitivity and may help reduce ectopic lipid accumulation in the liver and skeletal muscle, thereby enhancing whole-body metabolic flexibility [95,97]. At the cardiac level, these systemic adaptations are consistent with more efficient mitochondrial oxidation, activation of the AMPK-SIRT1-PGC-1α signaling axis, and reinforcement of Nrf2-mediated antioxidant defenses. In parallel, they may help attenuate NF-κB-driven inflammatory signaling, thereby limiting oxidative stress and chronic low-grade inflammation that promote adverse cardiac remodeling. [98]. Moreover, nutrient- and microbiota-derived metabolites that are typically enriched in plant-based diets, such as SCFAs, methyl-donor substrates, and polyphenols, may modulate epigenetic mechanisms (DNA methylation, histone acetylation, and non-coding RNAs) involved in the regulation of cardiac metabolism, potentially contributing to a cardioprotective form of “metabolic memory.” Although much of the epigenetic evidence still comes from preclinical models or indirect markers, the consistency between clinical endpoints (LDL-C, apoB, HbA1c, and body weight) and the engagement of these nutrient-sensing and epigenetic pathways supports the notion that well-planned vegetarian and vegan diets may represent high-intensity nutritional strategies that can complement pharmacological therapy in selected patients [99].

## 7. Microbiota, Metabolites, and the Heart

There is growing evidence that the gut microbiota is an important mediator of cardiovascular function in the context of diet-induced metabolic regulation. It functions as a dynamic metabolic interface rather than a passive ecosystem, influencing the availability of bioactive metabolites that influence the heart’s redox balance, mitochondrial function, and epigenetic regulation. Dysbiosis, a disorder that promotes systemic inflammation and contributes to the development of atherosclerosis, heart failure, and hypertension, results from a disruption of this balance [100,101]. Therefore, a functional gut–heart axis is defined by the interaction between diet and the gut microbiota, through which nutrients alter the molecular pathways that control inflammation, energy homeostasis, and cardiac remodeling.

### 7.1. The Role of the Intestinal Microbiota in Cardiac Metabolism

**Figure 6:**
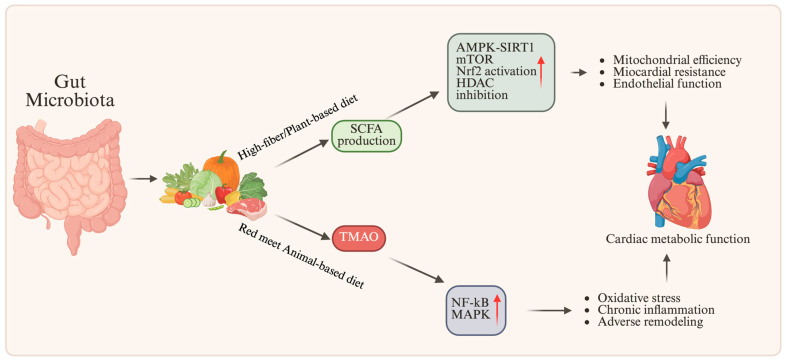
Gut microbiota-derived metabolites modulate cardiac metabolic function. A balanced diet promotes the production of SCFAs, which activate AMPK–SIRT1 signaling and improve mitochondrial efficiency, myocardial stress resistance, and endothelial function. In contrast, a diet rich in red meat and saturated fats increases TMAO levels, triggering NF-κB/MAPK pathways and promoting oxidative stress, chronic inflammation, and adverse cardiac remodeling. Arrows indicate the direction of pathway interactions, while red upward arrows (↑) indicate increased activation of the indicated signaling pathways. SCFAs (Short-chain Fatty Acids); TMAO (Trimethylamine N-oxide); AMPK (AMP-activated protein kinase); mTOR (Mammalian target of rapamycin); SIRT1 (Sirtuin1); HDAC (Histone deacetylase); NF-κB (Nuclear factor kappa-light-chain-enhancer of activated B cells); MAPK (Mitogen-activated protein kinase).

The gut microbiota is another regulatory axis linking diet to cardiac metabolic function, consistent with the nutritional regulation discussed previously. It improves mitochondrial efficiency, endothelial function, and myocardial resistance to oxidative stress by supporting AMPK-SIRT1-dependent pathways through the microbial conversion of nutrients into SCFAs such as acetate, propionate, and butyrate [102,103]. Conversely, by activating NF-κB and MAPK signaling, a diet high in red meat and saturated fat promotes the production of Trimethylamine N-oxide (TMAO), a metabolite linked to oxidative stress, chronic inflammation, and maladaptive remodeling [104,105]. Therefore, the microbiota’s metabolic output is determined by dietary patterns, which in turn influence redox balance and systemic energy homeostasis. A eubiotic microbial community, enriched in genera such as Faecalibacterium, Bifidobacterium, and Roseburia, is associated with improved metabolic flexibility and a cardioprotective phenotype. From a functional standpoint, two contrasting microbiota-related states can be broadly distinguished. These include an SCFA-dominant, cardioprotective microbiota profile and a TMAO-dominant profile associated with adverse cardiac remodeling. Figure 6 summarizes how differences in dietary substrate availability shape microbial metabolite profiles and downstream cardiac effects. In SCFA-dominant states, typically associated with high-fiber, plant-rich diets, the greater availability of fermentable substrates (e.g., dietary fiber, resistant starch, and complex carbohydrates) enhances saccharolytic fermentation and increases the production of acetate, propionate, and butyrate, which support mitochondrial efficiency, activate AMPK-SIRT1 signaling, enhance Nrf2-dependent antioxidant responses, and dampen NF-κB-mediated inflammation, thereby promoting metabolic flexibility and a cardioprotective phenotype [106,107]. In TMAO-dominant states, driven by high intake of animal-based sources of TMAO precursors (choline, phosphatidylcholine, and L-carnitine) in the context of dysbiosis, microbial conversion of these substrates into trimethylamine (TMA) and its hepatic oxidation to TMAO (via FMO3) are associated with endothelial dysfunction, platelet hyper-reactivity, fibrosis, and impaired cardiac remodeling [5,108,109].

### 7.2. Microbial Metabolites and Epigenetic Signaling

Metabolites produced by the gut microbiota influence cardiac gene expression through a variety of epigenetic mechanisms, expanding the concept of nutritional epigenetics. Within this framework, Table 6 provides a comparative overview of SCFA- versus TMAO-dominant microbiota-derived metabolic profiles, highlighting their distinct epigenetic mechanisms, associated signaling pathways, and translational implications for cardiac function. Butyrate, in particular, functions as a natural HDAC inhibitor, increasing histone acetylation and stimulating the transcription of genes related to antioxidant defense and mitochondrial respiration [76,77]. Gallic acid and urolithin A are examples of microbial-derived polyphenolic metabolites that control miRNA expression and DNA methylation, promoting SIRT1/PGC-1α-mediated pathways and preventing myocardial fibrosis [110,111]. On the other hand, TMAO impairs cardiac adaptability by suppressing Nrf2 transcription and increasing methylation of antioxidant gene promoters. All these findings indicate that diet is a potent epigenetic modulator capable of altering the way the microbiota and the heart interact [100]. The main diet-derived microbial metabolites and their epigenetic and functional effects on the heart are summarized in Table 6.

### 7.3. Nutritional Approaches Aimed at Modulating the Microbiota

Nutritional interventions that restore intestinal eubiosis have emerged as a promising frontier in cardiovascular prevention, converting molecular knowledge into actionable strategies. Diets rich in soluble fiber, polyphenols, and unsaturated fatty acids promote the growth of SCFA-producing bacteria, strengthening anti-inflammatory and antioxidant defenses that maintain cardiac and vascular health [112]. Conversely, excessive consumption of refined sugars and saturated fats weakens the integrity of the intestinal barrier and reduces microbial diversity. This allows lipopolysaccharides (LPS) to translocate throughout the body and activate Toll-like receptor 4 (TLR4) signaling, leading to endothelial dysfunction and chronic inflammation [113]. Targeted prebiotic and probiotic supplementation increases the production of cardioprotective metabolites and promotes positive epigenetic remodeling, especially when combined with balanced dietary patterns such as the Mediterranean diet [114]. All these findings support the idea that a key component of precision cardiometabolic medicine is modifying the gut–heart axis through diet. SCFAs and other gut-derived metabolites act as natural inhibitors of HDAC, providing a molecular pathway through which the microbiota and diet influence cardiac epigenetic remodeling [5,115].

## 8. Clinical Implications and Future Perspectives

The relationship between diet and cardiac function has been redefined in recent years by growing evidence, emphasizing the importance of nutrition as an active biological modulator rather than a simple metabolic variable. It is now known that dietary components are molecular signals capable of influencing the metabolic pathways that determine cardiac adaptation, gene expression, and epigenetic regulation [116,117]. The idea that nutrients function both as metabolic substrates and molecular effectors, activating intracellular pathways and altering gene transcription in a protective or detrimental manner, has been consolidated by the findings of nutriepigenetic research. Precision nutrition, a field that combines genetic, epigenetic, and metabolomic data to create personalized dietary plans aimed at improving cardiac function, increasing the body’s ability to adapt to oxidative and inflammatory stress, and reducing the risk of chronic cardiovascular disease, has been enabled by this paradigm shift [118]. Consequently, a systemic and preventive framework, in which diet emerges as a type of molecular therapy capable of modulating the mechanisms underlying cardiometabolic dysfunction, is gradually replacing the traditional clinical model, which focuses on managing isolated risk factors. Within the broader field of precision medicine, the integration of systems biology, clinical practice, and omics sciences is redefining cardiovascular prevention, shifting it toward a predictive and personalized paradigm [119,120].

### 8.1. Personalized Nutrition and Precision Medicine in Cardiology

**Figure 7:**
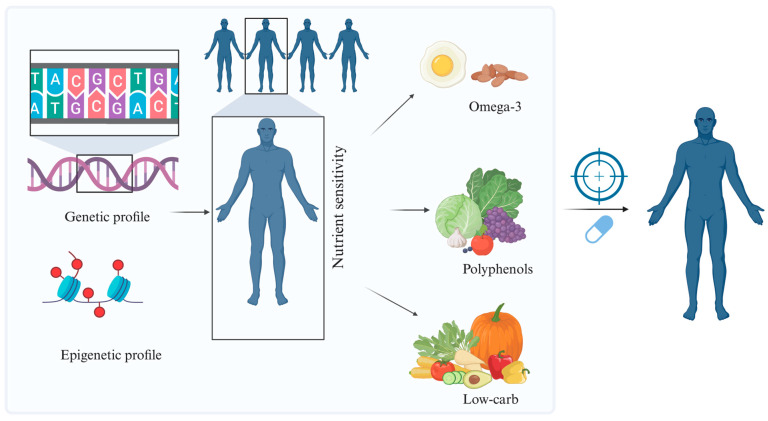
Influence of Genetic and Epigenetic Profiles on Nutrient Sensitivity. Schematic overview illustrating how individual genetic and epigenetic profiles influence sensitivity to specific nutrients, including omega-3 fatty acids, polyphenols, and low-carbohydrate diets. Variations in these molecular signatures modulate metabolic, inflammatory, and epigenetic responses, forming the basis for personalized nutritional strategies.

One of the most revolutionary developments in contemporary cardiology is the application of personalized nutrition. Genetic, epigenetic, and microbiota-dependent factors that influence the availability and utilization of nutrients are responsible for significant interindividual variability in metabolic responses to diet [121]. By combining genomic and epigenetic profiling, it is now possible to identify individuals with particular sensitivity to nutrients such as polyphenols, low-glycemic carbohydrates, and omega-3 fatty acids, improving nutritional and therapeutic outcomes [122] (Figure 7). With the help of artificial intelligence and multi-omics platforms, emerging precision nutrition models enable the personalization of interventions based on individual metabolic signatures and the prediction of dietary efficacy [123]. Looking forward, stratifying patients according to their epigenetic, metabolic, and inflammatory profiles will enable the design of targeted preventive and therapeutic protocols, with measurable impact on both prognosis and quality of life. Interindividual variability is a major determinant of cardiometabolic responses to nutritional interventions. Genetic background, pre-existing epigenetic marks, and baseline gut microbiota composition can all modulate how nutrient-sensing pathways, mitochondrial function, and epigenetic remodeling are engaged in a given individual. These factors help explain why similar dietary changes may yield heterogeneous effects on lipid profile, glycemic control, inflammatory status, or cardiac function across patients [124,125,126]. In this context, multi-omics approaches integrating genomics, epigenomics, transcriptomics, metabolomics, and metagenomics may refine risk stratification and support the tailoring of plant-based, Mediterranean, or nutraceutical strategies to specific molecular phenotypes [124]. However, multi-omics-guided personalization remains an emerging approach and its translation into routine cardiology practice faces key challenges, including protocol standardization, cost, data integration, interpretability, and the need for prospective trials specifically designed to test personalized nutrition.

### 8.2. Dietary Interventions Integrated with Pharmacotherapy and Lifestyle Changes

Combining pharmacological and behavioral strategies with nutritional interventions significantly increases their clinical impact. Increased cardioprotective effects and reduced oxidative and inflammatory stress are the result of the interaction between diet and drugs, which specifically modulate shared molecular pathways: AMPK, SIRT1, and mTOR [48,127]. The combination of a Mediterranean diet and statin therapy is an example of this interaction, as it has been shown to improve endothelial function and ventricular compliance by reducing circulating levels of TMAO and pro-inflammatory cytokines [118,128]. Similarly, metformin enhances the benefits of low-carbohydrate dietary habits by activating AMPK, which increases cardiac energy efficiency and metabolic flexibility. Viewed from a broader perspective, the integration of nutrition, pharmacotherapy, and lifestyle modification represents a multidimensional therapeutic strategy that not only prevents cardiovascular disease but also supports the recovery of metabolic adaptability and functional reversibility in the myocardium [129,130]. Table 7 summarizes the synergistic interactions among pharmacological agents, metabolic pathways, and dietary interventions.

### 8.3. Epigenetic Biomarkers and Translational Research

The identification of epigenetic biomarkers that reflect individual responses to pharmacological and nutritional interventions is a key goal of advances in cardiovascular precision medicine. Recent research identifies miRNA profiles, histone modifications, and DNA methylation signatures as dynamic markers of cardiac remodeling and metabolic adaptation to diet [131,132]. Current research is now examining the clinical translation of these markers as tools for early diagnosis, patient stratification, and therapy monitoring, rather than focusing on their mechanistic dimension. Dietary and pharmacological interventions can alter the epigenetic networks that control mitochondrial function and oxidative balance, as demonstrated by the targeted modulation of regulators such as SIRT1, AMPK, and HDACs [133,134]. The creation of flexible and patient-specific treatment plans will be made possible by integrating these biomarkers into multi-omics frameworks and AI-assisted predictive models, bridging molecular research with clinical practice. According to the selected data, this convergence represents a crucial step toward a translational model of precision nutrition that will make epigenetic profiling a practical component of cardiovascular prevention [120].

## 9. Distinction Between Mechanistic, Translational, and Clinical Evidence

The studies discussed in this review cover multiple levels of evidence, ranging from mechanistic experiments in cellular and animal models to translational and observational research in humans. Mechanistic studies provide detailed insights into nutrient-sensing pathways, mitochondrial dynamics, and epigenetic regulation; however, their translational relevance to human physiology may be limited by species-specific metabolic differences and highly controlled experimental conditions. Translational research, including early-phase human studies and integrative multi-omics analyses, offers an intermediate level of evidence by linking molecular mechanisms to physiological outcomes, but it often lacks the statistical power and long-term follow-up needed to demonstrate clinical benefit. Evidence of direct clinical applicability remains comparatively limited, as there are still relatively few robust randomized controlled trials evaluating the metabolic, epigenetic, and cardiovascular effects of specific dietary patterns. Explicitly distinguishing these tiers of evidence is therefore essential to avoid premature causal inferences and to more clearly define priorities for future research (Table 8).

## 10. Conclusions

According to available data, diet plays an important biological role in determining cardiac function through a complex network of metabolic, inflammatory, and epigenetic processes. Continuous communication between nutrients, microbiota, and intracellular signaling determines the myocardium’s ability to tolerate oxidative and energetic stress and shapes its metabolic adaptability. A balanced dietary pattern rich in unsaturated fats, complex carbohydrates, and bioactive nutrients has been associated with improvements in inflammatory status, redox balance, and markers of mitochondrial function, thereby supporting cardiometabolic stability. On the other hand, high-calorie diets rich in refined sugars and saturated fats have been linked to altered energy homeostasis and cause harmful epigenetic remodeling, potentially contributing to heart failure progression and cardiometabolic risk. Long-term dietary exposures may imprint relatively stable epigenetic signatures, creating a form of metabolic memory that could contribute to sustained cardioprotective adaptations [135]. By modulating AMPK, SIRT1, mTOR, and Nrf2 signaling, nutritional strategies such as CR, IF, the Mediterranean diet, and plant-based diets have shown favorable effects in mechanistic and translational models, with supportive, although heterogeneous, evidence in humans, restoring the balance between substrate oxidation, redox balance, and systemic inflammation. These strategies may induce epigenetic changes that support a cardioprotective metabolic phenotype; however, the durability of these signatures and their causal contribution to clinical outcomes in humans remain to be established [131]. At the same time, it should be acknowledged that much of the available evidence comes from heterogeneous observational cohorts and relatively small or short-term intervention studies, and that multi-omics and epigenetic approaches are still emerging and not yet fully standardized. In addition, variability in dietary assessment, adherence, background therapies, and the frequent reliance on surrogate endpoints limit causal inference and clinical generalizability. These aspects do not weaken the overall message of the review but rather highlight clear priorities for future, more harmonized mechanistic and clinical investigations. According to new data, personalized nutritional plans based on multi-omic profiles and epigenetic biomarkers represent a promising and rapidly evolving direction in precision cardiology. From this perspective, diet may be considered a modifiable, complementary intervention that could help inform prevention and risk reduction strategies, rather than being framed as a stand-alone molecular therapy at present. To translate mechanistic insights into evidence-based interventions, researchers, clinicians, and nutrition specialists must work closely to achieve this integration between molecular nutrition and clinical practice. Investing in the study of the metabolic and epigenetic relationships between nutrients and cardiac tissue will help define testable hypotheses and identify a patient’s subgroup and mitigation of cardiovascular disease. In conclusion, a personalized and multifaceted paradigm in which nutrition evolves into a molecular therapeutic tool that targets the biological causes of cardiac dysfunction could be a key to the future of cardiovascular prevention and treatment.

## Figures and Tables

**Table 1:** Signaling Pathways and Molecular Regulators of Cardiac Metabolism. Key signaling pathways and molecular regulators of cardiac metabolism, showing their roles, activating stimuli, main effects, experimental models, and consequences of dysfunction.

Pathway/ Regulator	Main Role	Stimuli/Activators	Main Effects	Models	Consequences of Dysfunction	References
PPARα	Regulates transcription of genes involved in fatty acid β-oxidation	Unsaturated fatty acids, ketone bodies	Enhances lipid utilization and prevents accumulation of lipotoxic intermediates	Human, animals, in vitro	Chronic exposure to saturated fats leads to metabolic inflexibility and cardiac dysfunction	[13]
AMPK	Principal cellular energy sensor (AMP/ATP ratio)	Increased AMP/ATP ratio, metabolic stress	Stimulates substrate oxidation, mitochondrial biogenesis, and antioxidant defenses	Animals, in vitro	Chronic caloric excess reduces its activity, impairing cardiac metabolic efficiency	[16]
mTOR	Nutrient-sensitive anabolic regulator	Nutrient overload *	Promotes protein synthesis and cell growth	Animals, in vitro	Sustained activation drives maladaptive cardiac hypertrophy; caloric restriction restores balance	[17]
SIRT1/PGC-1α	Integrates redox and energy pathways, regulates mitochondrial gene expression	Polyphenols, omega-3 fatty acids	Enhances oxidative efficiency and preserves cardiac function	Animals, in vitro	Mitochondrial dysfunction and loss of energetic homeostasis	[18]

PPARα (Peroxisome proliferator-activated receptor alpha); AMPK (AMP-activated protein kinase); mTOR (Mammalian target of rapamycin); SIRT1/PGC-1α (Sirtuin1/Peroxisome proliferator-activated receptor gamma coactivator 1-alpha); ATP (Adenosine triphosphate). * Nutrient overload is here intended as a chronic excess of total energy intake and specific macronutrients (particularly refined sugars and saturated/trans fats) relative to energy expenditure, leading to lipid and glucose accumulation, insulin resistance, and metabolic stress in cardiac tissue.

**Table 2:** Modulatory effects of bioactive nutrients on oxidative and inflammatory pathways. Effects of bioactive nutrients on oxidative stress and inflammatory pathways in the heart, including key signaling targets, functional outcomes, experimental models, and impacts on cardiac function.

Nutrient or Dietary Pattern	Main Pathways Modulated	Effect on Oxidative/Inflammatory Balance	Models	Cardiac Functional Impact	References
Saturated fats and simple sugars	↑ NF-κB, ↓ Nrf2	↑ ROS and cytokine production, reduced antioxidant capacity	Human, animals, in vitro	Fibrosis, reduced contractile reserve	[30,46]
Mediterranean diet	↑ AMPK, ↑ SIRT1	↓ inflammation, ↑ antioxidant response	Animals, in vitro	Improved endothelial and metabolic function	[48]
Polyphenols and omega-3 PUFA	↑ Nrf2, ↑ PPARα	↓ lipid peroxidation, ↓ NF-κB activation	Animals, in vitro	Enhanced energy efficiency, ↓ cardiac remodeling	[50]
CR	↑ AMPK	↓ ROS, ↑ mitochondrial efficiency	Human	Increased myocardial resilience and adaptive capacity	[51]

CR (Caloric restriction); NF-κB (Nuclear factor kappa-light-chain-enhancer of activated B cells); Nrf2 (nuclear factor erythroid 2-related factor 2); AMPK (AMP-activated protein kinase); SIRT1 (sirtuin 1); PPARα (Peroxisome proliferator-activated receptor alpha); ROS (Reactive oxygen species). ↑ indicates increase; ↓ indicates decrease.

**Table 3:** Epigenetic nutrients and metabolites with cardioprotective activity. Cardioprotective nutrients and metabolites, their epigenetic mechanisms, experimental models, and effects on cardiac function.

Nutrient/Dietary Component	Epigenetic Mechanism	Models	Functional Effect on the Heart	Typical Amount for Benefit	References
Folate ^1^, choline ^2^, methionine ^3^, betaine ^4^	DNA methylation via DNMT modulation and methyl-donor supply	Human, animals, in vitro	Restores proper methylation, enhances mitochondrial function, reduces lipid accumulation	400–800 µg/day ^1^, 425–550 mg/day ^2^, 13–15 mg/kg/day ^3^, 2–6 g/day ^4^	[57,58]
Saturated fats and simple sugars	Altered DNA methylation and miRNA expression	Human, animals, in vitro	Promotes fibrosis, apoptosis, oxidative stress, and metabolic inflexibility	Limit intake: <10% total daily energy from saturated fats; added sugars <25 g/day	[71,72]
SCFAs	DNMT and HDAC inhibition; modulation of methyl-donor availability	Human, animals, in vitro	Improves redox balance, enhances antioxidant gene expression, and supports gut–heart communication	3–5 g/day (from fiber)	[58]
Resveratrol (polyphenols)	SIRT1 activation and histone deacetylation	Animals, in vitro	Promotes mitochondrial biogenesis, improves oxidative stress resistance	150–500 mg/day	[61,62]
Omega-3 fatty acids	HDAC modulation and histone acetylation	Human, animals, in vitro	Reduces inflammation and enhances antioxidant capacity	1–3 g/day EPA + DHA	[73]
Sulfur compounds (e.g., garlic and cruciferous vegetables)	HDAC inhibition	Human, animals, in vitro	Enhances antioxidant defenses, reduces vascular inflammation	30–60 mg/day sulforaphane (from cruciferous vegetables), 5–10 mg/day allicin (from garlic)	[64]
Polyphenols and unsaturated fats (Mediterranean diet)	miRNA regulation (↑ miR-133a, miR-499; ↓ miR-21, miR-34a)	Human	Improves contractility, limits fibrosis and apoptosis	Diet-based: ~30–50 g olive oil, 200–300 g vegetables, 20–40 g nuts/day	[68,69,70,74]

SCFAs (Short-chain fatty acids); DNMT (DNA methyltransferase); HDAC (Histone deacetylase); SIRT1 (Sirtuin1); miRNA (microRNA); EPA (Eicosapentaenoic Acid); DHA (Docosahexaenoic Acid). Superscript numbers (^1–4^) indicate the corresponding typical amounts for benefit: ^1^ folate (400–800 µg/day), ^2^ choline (425–550 mg/day), ^3^ methionine (13–15 mg/kg/day), ^4^ betaine (2–6 g/day). ↑ indicates increase; ↓ indicates decrease.

**Table 4:** Comparative effects of Mediterranean and Plant-Based diets on cardiac metabolism and molecular pathways. Comparative overview of Mediterranean and plant-based diets, including key dietary components, their molecular and epigenetic mechanisms, experimental models, and resultant effects on cardiac metabolism, oxidative stress, inflammation, and overall myocardial function.

Diet Type	Main Components	Molecular Mechanisms	Models	Main Cardiac Effects	References
Mediterranean diet	Olive oil, fish, fruits, vegetables, whole grains, red wine	Activation of SIRT1/PGC-1α, Nrf2 upregulation, modulation of miRNA expression	Human	Reduced inflammation and oxidative stress, improved endothelial function and lipid metabolism	[84,85]
Plant-based diet	Legumes, whole grains, nuts, fruits, vegetables, soy products	Reduction in oxidative stress, modulation of SCFA and gut microbiota composition	Human	Lower CVD risk, enhanced insulin sensitivity, improved mitochondrial metabolism	[75]

SIRT1/PGC-1α (sirtuin 1/peroxisome proliferator-activated receptor gamma coactivator 1-alpha); Nrf2 (nuclear factor erythroid 2-related factor 2); SCFAs (Short-chain fatty acids).

**Table 5:** Nutritional adequacy considerations for vegetarian/vegan (plant-based) diets in cardiology practice. This box summarizes key micronutrient- and macronutrient-related issues that may arise in patients following vegetarian or vegan diets, together with main food sources, supplementation options, and practical clinical considerations.

Nutrient	Potential Issue in Vegetarian/Vegan Patients	Main Sources/Supplementation	Practical Clinical Considerations	References
Vitamin B12	High risk of deficiency and hyperhomocysteinemia, especially in vegans	Fortified foods, oral B12 supplements	Mandatory supplementation in vegans; often advisable in older adults and in patients on metformin or proton pump inhibitors; monitor serum B12 and homocysteine.	[92,93,94,95,96,99]
Iodine	Inadequate intake or, less frequently, excessive intake from seaweed	Iodized salt, seaweed (with caution), fortified foods	Recommend iodized salt; avoid excessive seaweed consumption in patients with thyroid disorders.	[95,97]
Selenium	Low intake in regions with selenium-poor soils	Brazil nuts, seeds, whole grains, low-dose supplements	Consider supplementation in low-selenium areas; interpret status in the context of overall diet and comorbidities.	[95,97]
Zinc	Mild deficiency risk, particularly with poorly varied plant-based diets	Legumes, seeds, nuts, whole grains	Encourage food preparation methods that enhance bioavailability (soaking, sprouting, fermentation).	[95,97]
Iron	Increased risk of deficiency in specific groups (e.g., premenopausal women)	Legumes, tofu, whole grains, seeds, leafy green vegetables	Combine with vitamin C-rich foods; monitor hemoglobin and ferritin in high-risk individuals.	[95,97]
Calcium	Potentially low intake, especially in strict vegan diets	Calcium-set tofu, leafy greens, mineral waters, fortified plant milks	Assess total intake; consider calcium-fortified foods if dietary intake is insufficient.	[95,97]
Vitamin D	Common insufficiency regardless of dietary pattern	Sun exposure, vitamin D supplements	In cardiac patients, assessment of serum 25(OH)D and supplementation is often warranted.	[86,91]
Omega-3 (EPA/DHA)	Low direct intake of EPA/DHA in the absence of fish	Flaxseed, chia, walnuts (ALA); microalgae-based EPA/DHA supplements	Consider microalgae-derived EPA/DHA in patients with established CVD or hypertriglyceridemia.	[73,86,91]
Protein quality	Suboptimal essential amino acid profile when plant proteins are not diversified	Legumes, soy foods, whole grains, nuts, seeds	Promote a varied combination of plant protein sources to ensure adequate total protein and essential amino acid intake.	[95,97,99]

CVD (cardiovascular disease); EPA (Eicosapentaenoic Acid); DHA (Docosahexaenoic Acid).

**Table 6:** SCFA- versus TMAO-dominant microbiota profiles. Comparative overview of SCFA- and TMAO-dominant microbiota-derived metabolic states and their epigenetic and cardiac implications.

Microbiota-Derived Profile	Predominant Dietary Pattern	Microbiota Functional State	Epigenetic Modulation	Key Signaling Pathways	Cardiac and Translational Effects	References
SCFA	Fiber-rich, Mediterranean, plant-based diets	Eubiotic, saccharolytic microbiota	HDAC inhibition; DNMT modulation → pro-adaptive epigenetic remodeling	↑ AMPK–SIRT1; ↑ Nrf2; ↓ NF-κB	Improved mitochondrial efficiency, reduced inflammation, enhanced metabolic flexibility	[76,77]
TMAO	Red meat-rich, high-fat diets	Dysbiotic, proteolytic microbiota	Increased DNA methylation of antioxidant genes → maladaptive epigenetic imprinting	↑ NF-κB; ↑ MAPK; ↓ Nrf2	Endothelial dysfunction, fibrosis, adverse remodeling, increased CVD risk	[100]
Microbial polyphenol metabolites (urolithin A, gallic acid)	Polyphenol-rich diets	Eubiotic	miRNA modulation; DNA hypomethylation	↑ SIRT1/PGC-1α	Anti-fibrotic effects, improved mitochondrial function	[110,111]

SCFAs (Short-chain Fatty Acids); TMAO (Trimethylamine N-oxide); HDAC (Histone deacetylase); DNMT (DNA methyltransferase); miRNA (microRNA); AMPK/SIRT1 (AMP-activated protein kinase/sirtuin1); Nrf2 (nuclear factor erythroid 2-related factor 2); NF-κB (Nuclear factor kappa-light-chain-enhancer of activated B cells); MAPK (Mitogen-activated protein kinase); SIRT1/PGC-1α (Sirtuin1/Peroxisome proliferator-activated receptor gamma coactivator 1-alpha); CVD (Cardiovascular disease).↑ indicates increase;↓ indicates decrease.

**Table 7:** Synergistic interactions between diet, pharmacotherapy, and metabolic pathways in cardioprotection. Synergistic interactions between dietary interventions and pharmacological treatments, highlighting involved metabolic pathways, molecular effects, experimental models, and resultant cardioprotective outcomes.

Combined	Main Pathways Involved	Molecular and Metabolic Effects	Models	Cardiovascular Outcomes	References
Mediterranean diet + statins	↑ AMPK, ↑ SIRT1, ↓ mTOR	↓ TMAO, improved endothelial function	Human	↑ ventricular compliance, ↓ atherosclerotic risk	[118,128]
Metformin + low-carbohydrate diet	↑ AMPK, enhanced mitochondrial oxidation	↑ cardiac energy efficiency, ↑ metabolic flexibility	Animals, In vitro	Improved exercise tolerance, ↓ oxidative stress	[130]
Polyphenol-rich diet + ACE inhibitors	↑ SIRT1, ↓ NF-κB	↓ inflammation and myocardial remodeling	Human, animals, in vitro	↓ fibrosis, ↑ diastolic function	[48]
Omega-3 PUFA + β-blockers	↑ PPARα, ↑ AMPK	↑ fatty acid utilization, ↓ plasma triglycerides	Human, animals, in vitro	↓ arrhythmias, ↑ cardiac electrical stability	[127]
Caloric restriction + moderate exercise	↓ mTOR, ↑ mitochondrial biogenesis	↑ metabolic resilience, ↓ ROS	Human, animals	Improved myocardial plasticity, ↓ ischemic events	[129]

AMPK (AMP-activated protein kinase); SIRT1 (Sirtuin1); mTOR (Mammalian target of rapamycin; NF-κB (Nuclear factor kappa-light-chain-enhancer of activated B cells); PPARα (Peroxisome proliferator-activated receptor alpha); ROS (Reactive oxygen species); TMAO (Trimethylamine N-oxide). ↑ indicates increase;↓ indicates decrease.

**Table 8:** Types and Strength of Evidence Supporting Nutritional and Epigenetic Regulation of Cardiac Metabolism. Summary of the types and strength of evidence (mechanistic, translational, clinical) supporting the effects of dietary patterns, nutrients, and epigenetic modulators on cardiac metabolism.

Dietary Pattern/Nutrient	Key Molecular Pathways	Type of Evidence	Strength of Evidence *	Notes/Limitations	References
CR	AMPK, SIRT1/PGC-1α, autophagy	Strong mechanistic; moderate translational; limited clinical	●●●○	Human evidence short-term only	[78,79,80,81,82,83,129]
IF	Ketone metabolism, AMPK, Nrf2	Strong mechanistic; emerging human observational	●●○○	Protocol heterogeneity	[80,81,82,83]
Mediterranean Diet	Anti-inflammatory, antioxidant pathways	Moderate observational; limited mechanistic	●●●○	Confounding difficult to control	[84,85,86,118]
Polyphenols	SIRT1 activation, histone acetylation	Strong mechanistic; weak clinical	●●○○	Bioavailability issues	[61,62,73]
Omega-3 Fatty Acids	Membrane signaling, anti-inflammatory	Moderate mechanistic; moderate clinical	●●●○	Dose and purity variation	[73,86,127]
SCFAs	HDAC inhibition, gut–heart axis	Emerging mechanistic	●○○○	Limited human data	[76,77,100]

SCFAs (Short-chain fatty acids); IF (Intermittent Fasting); CR (Calorie Restriction); AMPK (AMP-activated protein kinase); SIRT1/PGC-1α (sirtuin 1/peroxisome proliferator-activated receptor gamma coactivator 1-alpha); Nrf2 (nuclear factor erythroid 2-related factor 2); HDAC (Histone deacetylase). * Strength of Evidence indicates the overall robustness of available data: ●●●○ strong evidence from converging mechanistic and human studies; ●●○○ moderate or emerging evidence; ●○○○ preliminary evidence mainly from experimental models.

## Data Availability

No new data were created or analyzed in this study. Data sharing is not applicable to this article.

## Abbreviations

The following abbreviations are used in this manuscript:

Akt	Protein kinase A
AMPK	AMP-activated protein kinase
ATP	Adenosine triphosphate
CR	Calorie restriction
CVDs	Cardiovascular diseases
DNMT	DNA methyltransferase
FOXO3a	Forkhead box O3a
HAT	Histone acetyltransferase
HDAC	Histone deacetylase
IF	Intermittent fasting
LPS	Lipopolysaccharide
lncRNA	Long non-coding RNA
MAPK	Mitogen-activated protein kinase
mTOR	Mammalian target of rapamycin
miRNA	microRNA
NF-κB	Nuclear factor kappa-light-chain-enhancer of activated B cells
NO	Nitric oxide
Nrf2	Nuclear factor erythroid 2-related factor 2
PGC-1α	Peroxisome proliferator-activated receptor gamma coactivator 1-alpha
PI3K	Phosphoinositide 3-kinase
PPARα	Peroxisome proliferator-activated receptor alpha
ROS	Reactive oxygen species
SCFA	Short-chain fatty acid
SIRT1	Sirtuin 1
TET	Ten-Eleven Translocation
TLR4	Toll-like receptor 4
TMAO	Trimethylamine N-oxide
